# The arisal of data spaces: why I am excited and worried

**DOI:** 10.3389/fimmu.2024.1461361

**Published:** 2024-10-22

**Authors:** Liesbet M. Peeters

**Affiliations:** ^1^ University MS Center (UMSC), Hasselt-Pelt, Belgium; ^2^ Biomedical Research Institute (BIOMED), Hasselt University, Diepenbeek, Belgium; ^3^ Data Science Institute (DSI), Hasselt University, Diepenbeek, Belgium

**Keywords:** real-world data, European Health Data Space, secondary use of health data, collaborative research, data interoperability

## Abstract

This paper explores the significant role of real-world data (RWD) in advancing our understanding and management of Multiple Sclerosis (MS). RWD has proven invaluable in MS research and care, offering insights from larger and diverse patient populations. A key focus of the paper is the European Health Data Space (EHDS), a significant development that promises to change how healthcare data is managed across Europe. This initiative is particularly relevant to the MS community. The paper highlights various data initiatives, discussing their importance for those affected by MS. Despite the potential benefits, there are challenges and concerns, especially about ensuring that the growth of various data platforms remains beneficial for MS patients. The paper suggests practical actions for the global MS community to consider, aimed at optimizing the use of RWD. The emphasis of this discussion is on the secondary use of health data, particularly in the European context. The content is based on the author’s own experiences and interpretations, offering a personal yet informed view on using RWD to improve MS research and patient care.

## Introduction

The multiple sclerosis (MS) community is fortunate to have a longstanding and successful legacy of using real-world data (RWD, **Table 1**) to address complex clinical problems. RWD often reflects larger and more representative populations and therefore is specifically fit-for-purpose to investigate for example disease behavior in a real-world setting, validation of outcome measures, comparative effectiveness and long-term safety of therapies. Additionally, RWD plays a crucial role in enhancing patient advocacy by informing policies on employment, reimbursement of treatments and access to healthcare services, as well as supporting routine healthcare practices. A growing number of real-world MS databases and registries produce long-term outcome data from large cohorts of people with MS (1–3).

**Table 1:** Glossary **-** for the purpose of this paper, the following definitions of concepts and terminologies are introduced as follows:.

• **Data space**: Comprehensive term that captures various dimensions of data handling, from its storage and organization to its processing, access and analytical use. • **Real-World Data (RWD)**: Pragmatically defined as any data that is gathered in the context of standard care as opposed to data gathered in an experimental setting such as a randomized clinical trial. Examples include registry data and data collected and stored using electronic health records (EHR). Real-world-evidence (RWE) is defined as any evidence generated using RWD. • **Core dataset**: Set of variables that represent the common denominator across different initiatives and their accompanying (minimal) datasets. • **Common Data Model (CDM)**: Standardized representation of content, independent from a purpose or research question, combined with a defined common infrastructure. Its purpose is to enable collaborative analyses by providing a defined framework and structure. • **Primary use of health data**: When health data is used to deliver health care to the individual from whom it is collected. For example: an MRI measurement taken for the purpose of diagnosing MS. • **Secondary (re-)use of health data:** When (existing) health data, originally collected for a specific primary purpose, is used for alternative objectives or research that differs from the initiative reason for data collection. For example: data originally collected for patient care and treatment optimization is re-used to inform regulatory policies and decisions, potentially leading to improved treatment guidelines and enhanced patient safety in the MS patient community. • **Patient registries:** Organized systems that use observational methods to collect uniform data on a population defined by a particular disease, condition or exposure, and that is followed over time. • **Big data:** large datasets which may be complex, multi-dimensional, unstructured and heterogeneous, which are accumulating rapidly and which may be analyzed computationally to reveal patterns, trends, and associations (e.g. RWD (such as electronic health records, insurance claims data and data from patient registries), genomics, clinical trials, spontaneous adverse drug reaction reports, social media and wearable devices).

The heterogeneity in MS management across Europe, combined with the variability in data collection methods (different formats and data acquisition software systems used across various data sources and MS registries), presents significant challenges. These differences can impact the interpretation of RWD at scale. Despite these challenges, the research community has realized that combining data from diverse sources across the globe presents significant opportunities for advancing our understanding of MS. To manage the challenges associated with heterogeneity, strategies such as incorporating detailed information about the origin and specification of the source data, ensuring use of high-quality data, involving domain experts in interpreting results, and investing in data harmonization strategies are essential. These approaches have enabled the research community to turn these challenges into opportunities, as seen in initiatives like the Big Multiple Sclerosis Data Network (BMSD - bigmsdata.org) and the COVID-19 in MS Global Data Sharing initiative (GDSI).

BMSD is the largest real-world MS data network and brings together leading MS registries and databases to allow joint analyses of very large merged or federated sets of structured clinical data. It was initiated in 2014 and currently consists of the national MS registries of the Czech Republic (4), Denmark (5), France (6), Italy (7) and Sweden (8) as well as the international MSBase (9). The total number of MS patients in BMSD amounts to over 250,000. In recent years, the BMSD has led on several studies, yielding critical data-driven insights into MS treatment and progression. For example, they uncovered significant patterns in treatment management strategies (10) and disability progression in secondary progressive MS (11). GDSI was project led by the MS Data Alliance and MS International Federation in collaboration with a multitude of global partners (12). In March 2020, as COVID-19 spread, the demand for data on its impact on people with MS surged. Within months, 19 global partners shared data on over 10,000 people with MS, which helped update global advice for MS patients regarding COVID-19 (13–15).

While the MS community has made significant strides in utilizing RWD for research and patient care, several existing and emerging large-scale collaborative efforts across Europe – though not specific for MS – are set to profoundly impact how RWD is managed and utilized across various disease, including MS. In the following paragraphs, several of these key initiatives will be highlighted and explained in detail, focusing on their objectives, relevance to the MS community, and the potential benefits of engaging with them. These ‘highlighted initiatives’ represent transformative efforts that are shaping the future of healthcare data. However, while they offer exciting possibilities, they also present unique challenges. The subsequent discussion will explore these challenges and offer actionable recommendations to help the MS community effectively navigate this evolving landscape, mitigate risks, and maximize the opportunities these initiatives provide.

## Highlighted initiative 1: The European Health Data Space (EHDS) – a revolutionary legislative framework

The EHDS is set to revolutionize healthcare management across a wide spectrum of stakeholders. Europe has been making continuous efforts aiming at enhancing the harmonization and integration of health data, which is needed in order to be able to create a digitized and connected healthcare system, as foreseen in the EHDS regulation. The EHDS proposal aspires to (i) support individuals to take control of their own health data, (ii) support the use of health data for better healthcare delivery, better research, innovation and policy making and (iii) enables the EU to make full use of the potential offered by a safe and secure exchange, use and reuse of health data (16). Two projects, while differing in focus, collectively aspire to enhance the concrete implementation of the EHDS: TEHDAS and HealthData@EU. TEHDAS (Towards The European Health Data Space - tehdas.eu), running from February 2021 to July 2023, focused on developing principles for the secondary use of health data, emphasizing dialogue and engagement across stakeholders, and establishing governance models for cross-border cooperation. This project involved 25 European countries and numerous stakeholders in discussions about health data usage for research and policymaking. In contrast, the HealthData@EU Pilot (ehds2pilot.eu), launched in October 2022, is building a pilot infrastructure for the EHDS, focusing on infrastructure development, testing, and evaluation. Involving 17 partners, this project aims to connect data platforms, develop services for research project support, and provide guidelines for data standards and security.

## Highlighted initiative 2: DARWIN-EU – an initiative by the European Medicine Agency (EMA)

**Figure 1:**
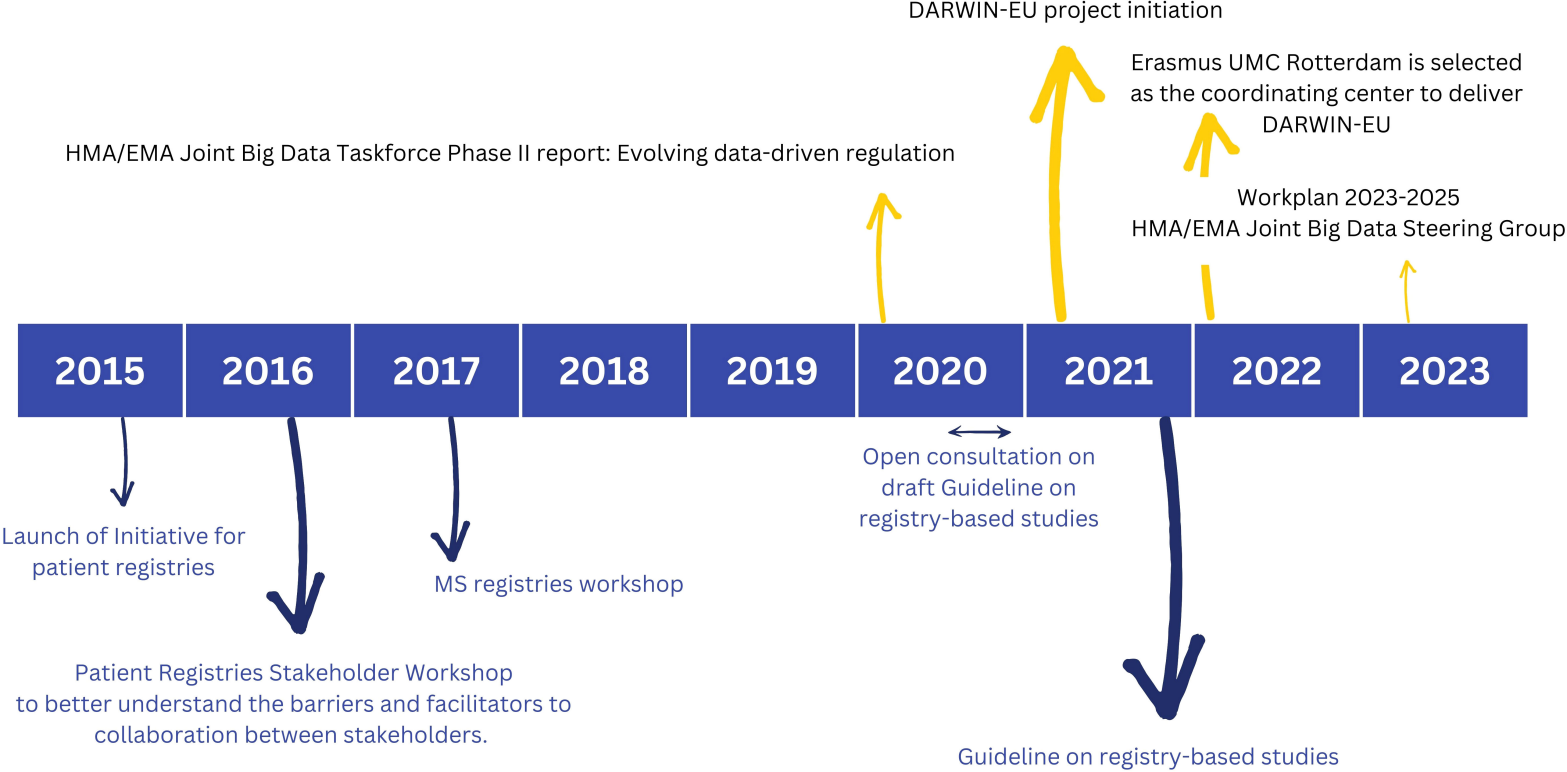
Highlighted activities of the European Medicine Agency (EMA) and/or the Heads of Medicine Agencies (HMA) with respective timelines focusing on ‘patient registries’ (bottom) and ‘big data’ (top). Reference documents to learn more include: Initiative for patient registries - Strategy and Mandate of the Cross-Committee Task Force (17); Guideline on registry-based studies (22); HMA/EMA Joint Big Data Taskforce Phase II report (23); Report - Patient Registry Workshop (18); Report on Multiple Sclerosis (MS) Registries (42): Work Plan 2023-2025 HMA/EMA Joint Big Data Steering Group (51).

The EMA has gained significant interest in the use of RWD to assess the benefit-risk of medicines across their lifecycle and to monitor the safety of medicine, specifically post-authorisation. A post-authorisation safety study (PASS) is a study that is carried out after a medicine has been authorized to obtain further information on a medicine’s safety, or to measure the effectiveness of risk-management measures. **Figure 1** highlights some of the key activities of EMA and/or the Heads of Medicine Agencies (HMA) with respective timelines.

The initiative for patient registries, launched in September 2015, aspired to explore ways of expanding the use of patient registries by introducing and supporting a systematic and standardized approach to their contribution to the benefit-risk evaluation of medicines (17). Within the scope of this initiative, two workshops of specific interest were hosted and summarized in extensive reports: (i) A more general disease-agnostic workshop on patient registries (2016) to better understand the barriers and facilitators to collaboration between stakeholders. The workshop report provides recommendations on actions to improve stakeholder collaboration and optimize the use of registries to support regulatory decision-making (18): (ii) An MS specific workshop aiming to reach consensus on implementable MS specific recommendations for advancing the systematic use of MS registries to support regulatory evaluations. Similar workshops were hosted for other disease registries such as for example haemophilia (19), cystic fibrosis (20) and cancer (21).

After a short period of public consultation, the guideline on registry-based studies was published in 2021. This guideline addresses the methodological, regulatory and operational aspects involved in using registry-based studies to support regulatory decision-making. It aims to help with defining study populations and designing study protocols. It provides guidance on data collection, data quality management and data analyses to achieve high quality evidence (22). Meta-data catalogues offering descriptive statistics will further support data quality assessment, and evolving guidelines on data quality criteria will continue to improve and standardize this process.

The HMA-EMA Joint Big Data Taskforce Phase II report (23) suggests how the European regulatory network can use Big Data to improve public health and innovation. The first and top priority activity formulated is to deliver a sustainable platform to access and analyze healthcare data from across the EU (Data Analysis and Real World Interrogation Network - DARWIN - darwin-eu.org). Other priority recommendations include to establish a framework for data quality and to enhance data discoverability by strengthening the current European Network of Centres for Pharmacoepidemiology and Pharmacovigilance (EnCePP) resources databases (24) in line with the ‘Good Practice Guide for the use of the Metadata Catalogue of RWD sources’ (25).

## Highlighted initiative 3: the Observational Medical Outcomes Partnership (OMOP) – driving data harmonization

The freely available OMOP (Observational Medical Outcomes Partnership) common data model (CDM) refers to the open community standardized data model, which is designed to integrate and harmonize healthcare data from various sources, such as electronic health records (EHRs), claims databases, and other observational databases (26, 27). The OMOP CDM is a patient-centric relational database with several standardized tables, distinguished in domains like condition, procedures, drug usage, measurements or observations. Some of the key standard terminologies used in the OMOP common data model include SNOMED CT ( (28) - snomed.org) and LOINC ( (29) - loinc.org). The large community behind the OMOP CDM is consolidated in the Observational Health Data Sciences and Informatics community (OHDSI - ohdsi.org). Some OHDSI tools of specific interest include HADES, a set of open source R-packages for large-scale analytics (30) and ATLAS, which facilitates the design and execution of analyses (31). The 2023 annual report on ohdsi.org highlighted impressive numbers: over 3,700 collaborators from 83 countries, a data network of 543 databases from 49 countries, and more than 956 million patient records, covering about 12% of the global population.

Several large-scale collaborative RWD initiatives have adopted the OMOP CDM. Some examples include PIONEER focusing on prostate cancer [prostate-pioneer.eu; (32)], the European Reference Network for Rare Adult Solid Cancers [EURACAN; euracan.eu; (33)], and HONEUR with a specific focus on hematology [portal.honeur.org; (34)]. The European Health Data and Evidence Network [EHDEN; ehden.eu; (35)] deserves special attention, since it managed to establish the largest European federated RWD network. The EHDEN network currently consists of 187 Data Partners in 29 countries across the European region, with greater than 850 million anonymous health records.

## Highlighted initiatives 4: European Research Data infrastructures: EBRAINS focusing on brain-related research data and ELIXIR for life sciences (-omics) data

Complementing these efforts are European research data infrastructures like EBRAINS (ebrains.eu) and ELIXIR (elixir-europe.org), which enhance research data handling and analysis for brain-related and life sciences (-omics) data, respectively. ELIXIR unifies bioinformatics resources and life science data for easier mining and reuse. This distributed digital infrastructure connects scientists from 23 countries (>250 research institutes), offering services like data deposition databases, data analysis, management, and compute services. ELIXIR also operates a vibrant training network through the TeSS Training Portal (36), registering over 1,200 training materials and training more than 19,000 people between September 2015 and March 2019 (37). ELIXIR played a leading role in the beyond one million genome project (b1mg-project.eu) that recently ended. During the COVID-19 pandemic, ELIXIR provided a range of services to study COVID-19 (38).

EBRAINS offers a digital infrastructure to boost collaborative brain research in neuroscience, brain health, and brain-related technology. Emerging from the Human Brain Project (HBP) (2013–2023), a European Flagship project with a €607 million investment, it involved over 500 researchers from 19 countries and 155 institutions. The HBP developed 160+ digital tools for multi-scale brain research and facilitated extensive collaboration among research teams (39). Some highlighted examples of potentially interesting tools and services include the Knowledge Graph - multi-modal metadata platform, the Medical Informatics Platform (MIP) - enabling access and analyses of anonymized medical data (40) and The Virtual Brain - a reference tool for full-brain simulation (41).

## Discussion

### There is great promise for the MS community in aligning closely and promptly with the EHDS legislation and engaging with emerging large-scale data initiatives that are not specific to MS

The EHDS is about to be implemented and is expected to have as significant and far-reaching impact. A proactive approach, which includes early investigation of alignment and synergy, would enable the MS community to understand the potential risks and challenges associated with this new legislation from the start. This foresight would allow for more effective long-term planning, the ability to anticipate future trends, and the development of risk management strategies to navigate anticipated changes in the regulatory environment. Moreover, collaborations with data initiatives not specific to MS not only pave the way for valuable partnerships and networking opportunities, but they also offer significant opportunities to explore new research questions and enhance existing studies with complementary insights.

### Nevertheless, the path forward is marked by numerous, significant challenges that need to be addressed

Although I am a firm advocate for the EHDS and the collaboration with the previously mentioned data initiatives, I must highlight a series of challenges and lingering questions. These will be summarized in the following section, underlining the complexities we still need to navigate:

- *How will the implementation of the EHDS impact the current utilization of MS registries and other RWD sources?* As previously emphasized, MS registries and other RWD sources are vital for addressing pressing clinical questions related to MS. Currently, there is significant variation in the governance principles applied within the existing and emerging registries and RWD sources, which complicates collaborative efforts (2, 42). Given the uncertainty regarding how the EHDS will influence the conduct of large-scale, multi-centric studies using data from different member states, it is yet to be determined whether the EHDS will simplify or further complicate these collaborations.
- *The EHDS primarily focuses on Europe, while other continents are advancing parallel initiatives within their regions, such as the Sentinel Initiative* (43)) *and the Framework for the FDA’s Real-World Evidence Program* (44)*. This raises the question: Can we expect alignment between these initiatives to address clinical challenges on a global scale?* Investigating phenomena like silent progression, pediatric MS, early detection of MS onset in at-risk individuals (referred to as prodrome), and conducting large-scale epidemiological studies, such as the Atlas of MS (45), requires a wealth of high-quality data. Global collaboration is crucial to tackle these complex questions, especially considering the global prevalence and incidence rates of MS. An estimated 2.8 million people worldwide live with MS, equating to 35.9 per 100,000 population, with a pooled incidence rate of 2.1 per 100,000 persons/year (45). For instance, the COVID-19 in MS global data sharing initiative brought together data from 19 partners but compiled ‘only’ 10,000 patient records (13–15). Similarly, the BMSD network, with the potential of over 250,000 patient records, experiences a significant reduction in numbers when specific inclusion criteria are applied (11).
- *How can we ensure that the disease-agnostic recommendations, services, and tools are not only fit-for-purpose but also implementable for addressing MS-related questions, given that their straightforward application to the MS community is evidently not as feasible as assumed?* A prime example is the OMOP CDM, which, despite its broad application, is currently not entirely suitable for MS registry data. This statement is based on the experiences of my research group and in line with the documented experience from pulmonary hypertension databases (46). The underlying problem and probably the main reason for the different mapping designs is the observational character of MS RWD sources that are not connected to an electronic health record and filled with clinical data from there. Furthermore, a significant gap exists between guidelines formulated by EMA and their practical application, as highlighted by two key reports – the EMA Report on MS Registries (18) and the EMA Guideline on Registry-Based Studies (22). These documents, while authoritative, lack the necessary detail, have little or no focus on patient’s input or patient relevant outcome measures and have not been checked sufficiently for real-world and sustainable implementation. For example, the discussion about financial sustainability is insufficiently incorporated into these reference documents. Despite the aforementioned challenges, there are notable examples of successful collaborations. The German MS registry and the MS DataConnect Cohort of the University MS Center in Belgium are part of the federated data network of EHDEN (35). In the MultipleMS consortium (multiplems.eu), linked to the International Multiple Sclerosis Genetics Consortium (47), and the COVID-19 in MS global data sharing initiative (12), the ELIXIR community has played a key role in supporting the technical architectures for data storage, management, and sharing in these large-scale collaborative efforts.

### In a continuously changing and complex environment, it is essential to prioritize pragmatic actions.

**Figure 2:**
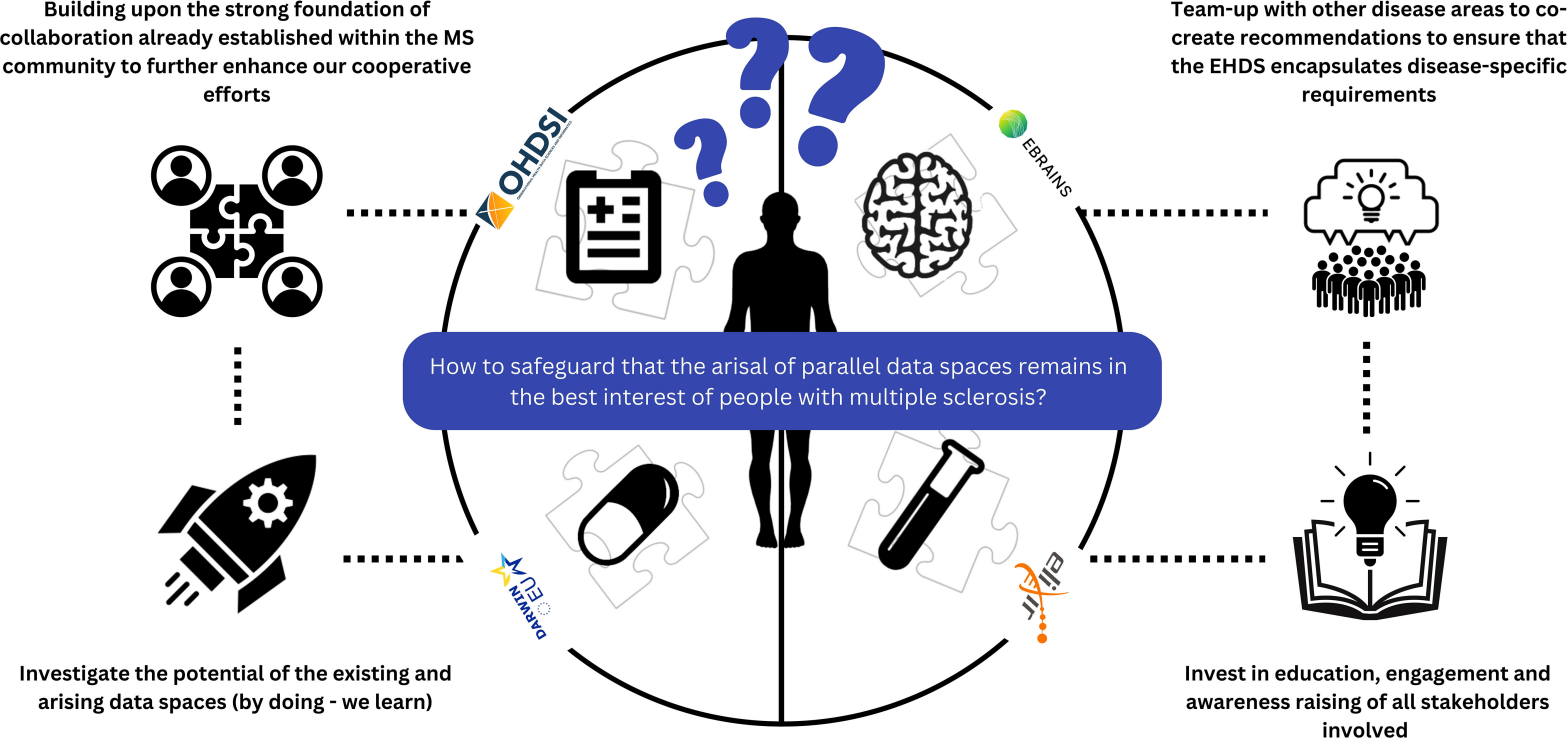
Summary overview of suggested action points towards the MS community to safeguard that the arisal of parallel data spaces remains in the best interest of people with multiple sclerosis.

To this end, a set of concrete, actionable suggestions for the MS community are formulated (see also **Figure 2**).

- Suggested action 1: Building upon the strong foundation of collaboration established within the MS community to further enhance our collaborative efforts. As we move toward formulating detailed and implementable global recommendations for data collection, it is clear that the responsibility for this initiative will continue to rest with the MS community. Recently, a global multi-stakeholder task force defined a core dataset for MS to guide emerging registries in their dataset definitions and speed-up and support harmonization across registries and RWD MS initiatives. A regular revision of the current Core DataSet is anticipated, especially in regards to the currently excluded variables or pragmatic choices of values (48). Dataset variables needing a dedicated set of data elements (e.g. in the area of patient-reported outcomes or pharmacovigilance) are also not included. The latter is anticipated to be driven by leading networks like BMSD or PROMS initiative focusing on these specific topics. Another interesting activity to enhance multi-stakeholder collaboration is to regularly organize large-scale multi-stakeholder engagement meetings (18, 49).
- Suggested action 2: Investigate the potential of existing and emerging data spaces to address some urgent and critical questions formulated by the MS community, adhering to the principle of ‘learning by doing.’ Specific pilot projects could be established and carried out to assess the suitability of current recommendations for data standardization, interoperability, infrastructure, and governance in the MS context. Following these pilot projects, identifying areas for potential synergy and proposing necessary adjustments will be crucial. An innovative approach could involve organizing a study-a-thon in collaboration with OHDSI and/or EHDEN. A study-a-thon is a focused, multi-day research event that generates reliable evidence on a specific medical topic across different countries and health systems. It gathers multidisciplinary teams to expedite scientific contributions without sacrificing the quality of research, facilitated through a reproducible process (50). This method could effectively showcase the advantages of collaborating with these networks within a limited timeframe. Concurrently, the MS Data Alliance is investigating how the OMOP CDM can be tailored to address the challenges previously identified. This research is specifically focused on the feasibility of automatically converting the MS Data Alliance Core Dataset (48) to the OMOP CDM, with the results expected to be publicly and freely available to the MS community soon.
- Suggested action 3: Team-up with other disease areas to co-2create recommendations to ensure that the EHDS encapsulates disease-specific requirements. The challenges highlighted earlier in this paper, while focusing on MS, are not unique to it. Similar issues are encountered by communities studying chronic diseases that require long-term, high-dimensional follow-up. Particularly relevant are those groups already actively engaged in EHDS discussions, such as those focused on cystic fibrosis, cancer and diabetes (20, 21, 49)). A practical first step would be to co-create a joint statement, consolidating a unified response to the EHDS proposal and addressing the identified challenges.
- Suggested action 4: Invest in education, engagement and awareness raising of all stakeholders involved to ensure proper understanding related to the EHDS as well as general data science principles. Stakeholders include regulators, clinicians, researchers, industry, and people with MS, all of whom are equally important. The level of being informed about how to contribute to the RWD ecosystem as well as experience in actively participating in large-scale RWD collaborative initiatives differs between stakeholders and individuals. Being limited informed and/or having limited experience leads to reduced active participation in initiatives that aim to address the urgent needs within the ecosystem. People with MS (or broader citizens) can actively contribute by co-creating legislation — deciding what is acceptable, how, and for what health data can be used - as well as helping to define priorities in the global research agenda.

## Conclusion

Rapid advances in artificial intelligence (AI) and the growing health data volume are expected to significantly impact the health sector. AI has already shown promise in helping to improve diagnostic performances, workflow and cost-effectiveness. AI has the potential to speed-up the complex process of data management and –analysis, specifically with the recent developments in the field of generative AI (e.g. ChatGPT). As we stand at the intersection of immense potential and complex challenges, there is both a reason for excitement and a cause for concern. By coming together – researchers, clinicians, patients, policymakers, and other stakeholders – we can harness the full potential of RWD while navigating its complexities. This is a journey that we must embark on together, informed by diverse perspectives and united by a common goal: to revolutionize MS care and research for the betterment of people affected by MS worldwide. Let this paper be the catalyst for that collaborative journey.

## Data Availability

The original contributions presented in the study are included in the article/supplementary material. Further inquiries can be directed to the corresponding author.

## References

[1] Trojano M Tintore M Montalban X Hillert J Kalincik T Iaffaldano P. Treatment decisions in multiple sclerosis — insights from real-world observational studies. Nat Rev Neurol. (2017) 13:105–18. doi: 10.1038/nrneurol.2016.188 28084327

[2] Geys L Parciak T Pirmani A McBurney R Schmidt H Malbaša T. The multiple sclerosis data alliance catalogue. Int J MS Care. (2021) 23:261–8. doi: 10.7224/1537-2073.2021-006 PMC8745233 35035297

[3] Cohen JA Trojano M Mowry EM Uitdehaag BM Reingold SC Marrie RA. Leveraging real-world data to investigate multiple sclerosis disease behavior, prognosis, and treatment. Mult Scler J. (2020) 26:23–37. doi: 10.1177/1352458519892555 PMC6950891 31778094

[4] Stastna D Drahota J Lauer M Mazouchova A Menkyova I Adamkova J. The Czech National MS Registry (ReMuS): Data trends in multiple sclerosis patients whose first disease-modifying therapies were initiated from 2013 to 2021. BioMed Pap. (2023) 168(3):262–70. doi: 10.5507/bp.2023.015.html 37114703

[5] Koch-Henriksen N Stenager E Brønnum-Hansen H. Studies based on the Danish multiple sclerosis registry. Scand J Public Health. (2011) 39:180–4. doi: 10.1177/1403494811405097 21775380

[6] Vukusic S Casey R Rollot F Brochet B Pelletier J Laplaud DA. Observatoire Français de la Sclérose en Plaques (OFSEP): A unique multimodal nationwide MS registry in France. Mult Scler J. (2020) 26:118–22. doi: 10.1177/1352458518815602 30541380

[7] on behalf of the Italian Multiple Sclerosis Register Centers Group Trojano M Bergamaschi R MP A Comi G Ghezzi A. The Italian multiple sclerosis register. Neurol Sci. (2019) 40:155–65. doi: 10.1007/s10072-018-3610-0 PMC6828204 30815752

[8] Hillert J Stawiarz L. The Swedish MS registry – clinical support tool and scientific resource. Acta Neurol Scand. (2015) 132:11–9. doi: 10.1111/ane.2015.132.issue-S199 PMC4657484 26046553

[9] Kalincik T Butzkueven H. The MSBase registry: Informing clinical practice. Mult Scler J. (2019) 25:1828–34. doi: 10.1177/1352458519848965 31120376

[10] Hillert J Magyari M Soelberg Sørensen P Butzkueven H van der Welt A Vukusic S. Treatment switching and discontinuation over 20 years in the big multiple sclerosis data network. Front Neurol. (2021) 12:647811. doi: 10.3389/fneur.2021.647811 33815259 PMC8010264

[11] Signori A Lorscheider J Vukusic S Trojano M Iaffaldano P Hillert J. Heterogeneity on long-term disability trajectories in patients with secondary progressive MS: a latent class analysis from Big MS Data network. J Neurol Neurosurg Psychiatry. (2023) 94:23–30. doi: 10.1136/jnnp-2022-329987 36171104

[12] Peeters LM Parciak T Walton C Geys L Moreau Y De Brouwer E. COVID-19 in people with multiple sclerosis: A global data sharing initiative. Mult Scler J. (2020) 26:1157–62. doi: 10.1177/1352458520941485 PMC7361123 32662757

[13] Simpson-Yap S Pirmani A Kalincik T De Brouwer E Geys L Parciak T. Updated results of the COVID-19 in MS global data sharing initiative: anti-CD20 and other risk factors associated with COVID-19 severity. Neurol Neuroimmunol Neuroinflamm. (2022) 9:e200021. doi: 10.1212/NXI.0000000000200021 36038263 PMC9423711

[14] Simpson-Yap S De Brouwer E Kalincik T Rijke N Hillert JA Walton C. Associations of disease-modifying therapies with COVID-19 severity in multiple sclerosis. Neurology. (2021) 97:1870–85. doi: 10.1212/WNL.0000000000012753 PMC8601210 34610987

[15] Simpson-Yap S Pirmani A De Brouwer E Peeters LM Geys L Parciak T. Severity of COVID19 infection among patients with multiple sclerosis treated with interferon-β. Mult Scler Relat Disord. (2022) 66:104072. doi: 10.1016/j.msard.2022.104072 35917745 PMC9310378

[16] Proposal for a regulation - The European Health Data Space - European Commission. Available online at: https://health.ec.europa.eu/publications/proposal-regulation-european-health-data-space_en (Accessed on July 8, 2024).

[17] EMA. Patient registry initiative-strategy and mandate of the cross-committee task force. EMA. Initiative London (2017).

[18] EMA. Patient registries workshop, 28 October 2016-observations and recommendations arising from the workshop. London: EMA (2017).

[19] EMA. Report on haemophilia registries workshop 8 june 2018(2018). Available online at: https://www.ema.europa.eu/en/documents/report/report-haemophilia-registries-workshop_en.pdf (Accessed on July 8, 2024).

[20] EMA. Report on Cystic Fibrosis Registries - Workshop 14 June 2017. London: EMA. (2017).

[21] EMA. Report of the workshop on the use of registries in the monitoring of cancer therapies based on tumours’ genetic and molecular features - 29 November 2019(2020). Available online at: https://www.ema.europa.eu/system/files/documents/report/report-workshop-registries_en.pdf (Accessed on July 8, 2024).

[22] Guideline on registry-based studies - Scientific guideline. European Medicines Agency. Available at: https://www.ema.europa.eu/en/guideline-registry-based-studies-scientific-guideline.

[23] Taskforce H. Phase II report:’evolving data-driven regulation. Eur Med Agency. (2019).

[24] Plueschke K Jonker C Strassmann V Kurz X. Collection of data on adverse events related to medicinal products: A survey among registries in the ENCePP resources database. Drug Saf.=. (2022) 45:747–54. doi: 10.1007/s40264-022-01188-x PMC9296377 35729468

[25] EMA EMA. Good Practice Guide for the use of the Metadata Catalogue of Real-World Data Sources(2022). Available online at: https://www.ema.europa.eu/en/documents/regulatory-procedural-guideline/good-practice-guide-use-metadata-catalogue-real-world-data-sources_en.pdf (Accessed on July 8, 2024).

[26] OMOP common data model. Available online at: https://ohdsi.github.io/CommonDataModel/ (Accessed on July 8, 2024).

[27] Voss EA Makadia R Matcho A Ma Q Knoll C Schuemie M. Feasibility and utility of applications of the common data model to multiple, disparate observational health databases. J Am Med Inform Assoc. (2015) 22:553–64. doi: 10.1093/jamia/ocu023 PMC4457111 25670757

[28] Spackman KA Campbell KE Côté RA. SNOMED RT: a reference terminology for health care. Proc Conf Am Med Inform Assoc AMIA Fall Symp. (1997), 640–4. PMC2233423 9357704

[29] Drenkhahn C Ingenerf J. The LOINC content model and its limitations of usage in the laboratory domain. Stud Health Technol Inform. (2020) 270:437–42. doi: 10.3233/SHTI200198 32570422

[30] OHDSI/Hades. Observational health data sciences and informatics(2024). Available online at: https://github.com/OHDSI/Hades (Accessed on July 8, 2024).

[31] OHDSI/Atlas. Observational health data sciences and informatics(2024). Available online at: https://github.com/OHDSI/Atlas (Accessed on July 8, 2024).

[32] Omar MI Roobol MJ Ribal MJ Abbott T Agapow PM Araujo S. Introducing PIONEER: a project to harness big data in prostate cancer research. Nat Rev Urol. (2020) 17:351–62. doi: 10.1038/s41585-020-0324-x 32461687

[33] Blay JY Casali P Bouvier C Dehais C Galloway I Gietema J. European Reference Network for rare adult solid cancers, statement and integration to health care systems of member states: a position paper of the ERN EURACAN. ESMO Open. (2021) 6:100174. doi: 10.1016/j.esmoop.2021.100174 34139485 PMC8219752

[34] Bardenheuer K Van Speybroeck M Hague C Nikai E Price M. Haematology Outcomes Network in Europe (HONEUR)—A collaborative, interdisciplinary platform to harness the potential of real-world data in hematology. Eur J Haematol. (2022) 109:138–45. doi: 10.1111/ejh.v109.2 35460296

[35] Voss EA Blacketer C Van Sandijk S Moinat M Kallfelz M Van Speybroeck M. European Health Data & Evidence Network—learnings from building out a standardized international health data network. J Am Med Inform Assoc. (2023) 31:209–19. doi: 10.1093/jamia/ocad214 PMC10746315 37952118

[36] Beard N Bacall F Nenadic A Thurston M Goble CA Sansone SA. TeSS: a platform for discovering life-science training opportunities. Bioinformatics. (2020) 36:3290–1. doi: 10.1093/bioinformatics/btaa047 PMC7214044 32044952

[37] Harrow J Drysdale R Smith A Repo S Lanfear J Blomberg N. ELIXIR: providing a sustainable infrastructure for life science data at European scale. Bioinformatics. (2021) 37:2506–11. doi: 10.1093/bioinformatics/btab481 PMC8388016 34175941

[38] Blomberg N Lauer KB. Connecting data, tools and people across Europe: ELIXIR’s response to the COVID-19 pandemic. Eur J Hum Genet. (2020) 28:719–23. doi: 10.1038/s41431-020-0637-5 PMC7225634 32415272

[39] Lorents A Colin ME Bjerke IE Nougaret S Montelisciani L Diaz M. Human brain project partnering projects meeting: status quo and outlook. eneuro. (2023) 10:ENEURO.0091–23.2023. doi: 10.1523/ENEURO.0091-23.2023 PMC10481639 37669867

[40] Redolfi A De Francesco S Palesi F Galluzzi S Muscio C Castellazzi G. Medical informatics platform (MIP): A pilot study across clinical Italian cohorts. Front Neurol. (2020) 11:1021. doi: 10.3389/fneur.2020.01021 33071930 PMC7538836

[41] Jirsa V Wang H Triebkorn P Hashemi M Jha J Gonzalez-Martinez J. Personalised virtual brain models in epilepsy. Lancet Neurol. (2023) 22:443–54. doi: 10.1016/S1474-4422(23)00008-X 36972720

[42] EMA. Report on multiple sclerosis registries - workshop 7 July 2017(2017). Available online at: https://www.ema.europa.eu/system/files/documents/report/wc500236644_en.pdf (Accessed on July 8, 2024).

[43] Brown JS Mendelsohn AB Nam YH Maro JC Cocoros NM Rodriguez-Watson C. The US Food and Drug Administration Sentinel System: a national resource for a learning health system. J Am Med Inform Assoc JAMIA. (2022) 29:2191–200. doi: 10.1093/jamia/ocac153 PMC9667154 36094070

[44] Schurman B. The framework for FDA’s real-world evidence program. (2019).

[45] Walton C King R Rechtman L Kaye W Leray E Marrie RA. Rising prevalence of multiple sclerosis worldwide: Insights from the Atlas of MS, third edition. Mult Scler J. (2020) 26:1816–21. doi: 10.1177/1352458520970841 PMC7720355 33174475

[46] Biedermann P Ong R Davydov A Orlova A Solovyev P Sun H. Standardizing registry data to the OMOP Common Data Model: experience from three pulmonary hypertension databases. BMC Med Res Methodol. (2021) 21:238. doi: 10.1186/s12874-021-01434-3 34727871 PMC8565035

[47] International Multiple Sclerosis Genetics Consortium (IMSGC) Beecham AH Patsopoulos NA Xifara DK Davis MF Kemppinen A. Analysis of immune-related loci identifies 48 new susceptibility variants for multiple sclerosis. Nat Genet. (2013) 45:1353–60. doi: 10.1038/ng.2770 PMC3832895 24076602

[48] Parciak T Geys L Helme A van der Mei I Hillert J Schmidt H. Introducing a core dataset for real-world data in multiple sclerosis registries and cohorts: Recommendations from a global task force. Mult Scler J. (2023) 30:13524585231216004. doi: 10.1177/13524585231216004 PMC10935622 38140852

[49] Hogervorst MA Møllebæk M Vreman RA Lu TA Wang J De Bruin ML. Perspectives on how to build bridges between regulation, health technology assessment and clinical guideline development: a qualitative focus group study with European experts. BMJ Open. (2023) 13:e072309. doi: 10.1136/bmjopen-2023-072309 PMC10462958 37640462

[50] Hughes N Rijnbeek PR van Bochove K Duarte-Salles T Steinbeisser C Vizcaya D. Evaluating a novel approach to stimulate open science collaborations: a case series of “study-a-thon” events within the OHDSI and European IMI communities. JAMIA Open. (2022) 5:ooac100. doi: 10.1093/jamiaopen/ooac100 36406796 PMC9670330

[51] EMA. Big Data Workplan 2023-2025 - HMA/EMA joint Big Data Steering Group (2024). Available online at: https://www.ema.europa.eu/en/documents/work-programme/workplan-2023-2025-hma-ema-joint-big-data-steering-group_en.pdf (Accessed on July 8, 2024).

